# The effect of the electrical double layer on hydrodynamic lubrication: a non-monotonic trend with increasing zeta potential

**DOI:** 10.3762/bjnano.8.152

**Published:** 2017-07-25

**Authors:** Dalei Jing, Yunlu Pan, Xiaoming Wang

**Affiliations:** 1 School of Mechanical Engineering, University of Shanghai for Science and Technology, Shanghai, 200093, P. R. China; 2 Key Laboratory of Micro-Systems and Micro-Structures Manufacturing, Ministry of Education and School of Mechatronics Engineering, Harbin Institute of Technology, Harbin, 150001, P. R. China,; 3 School of Electrical Engineering and Automation, Harbin Institute of Technology, Harbin, 150001, P. R. China

**Keywords:** electrical double layer, electroviscous effect, hydrodynamic lubrication, zeta potential

## Abstract

In the present study, a modified Reynolds equation including the electrical double layer (EDL)-induced electroviscous effect of lubricant is established to investigate the effect of the EDL on the hydrodynamic lubrication of a 1D slider bearing. The theoretical model is based on the nonlinear Poisson–Boltzmann equation without the use of the Debye–Hückel approximation. Furthermore, the variation in the bulk electrical conductivity of the lubricant under the influence of the EDL is also considered during the theoretical analysis of hydrodynamic lubrication. The results show that the EDL can increase the hydrodynamic load capacity of the lubricant in a 1D slider bearing. More importantly, the hydrodynamic load capacity of the lubricant under the influence of the EDL shows a non-monotonic trend, changing from enhancement to attenuation with a gradual increase in the absolute value of the zeta potential. This non-monotonic hydrodynamic lubrication is dependent on the non-monotonic electroviscous effect of the lubricant generated by the EDL, which is dominated by the non-monotonic electrical field strength and non-monotonic electrical body force on the lubricant. The subject of the paper is the theoretical modeling and the corresponding analysis.

## Introduction

As one of the oldest techniques in modern engineering, lubrication is widely recognized and has inspired significant scientific interest [1–4]. The use of a layer of lubricant film, either in solid or fluid state, between frictional pairs can effectively reduce friction and wear. With respect to fluid lubrication, it can be divided into various regimes or states based on the thickness of the lubricant film, such as dry friction with a lubricant film thickness of ≈1–10 nm, boundary lubrication (≈1–50 nm), thin film lubrication (≈10–100 nm) and fluid film lubrication (≈0.1–100 μm) [3,5]. Considering the small dimension of the fluid lubrication film (on the micro/nanoscale in the thickness direction), the chemical and physical properties of the solid–lubricant interface play an important role on the lubrication. Among these interfacial properties, the effect of charged frictional pair–lubricant interface and the resulting electrical double layer (EDL) within the lubricant have been recognized and studied [5–13]. The generation of surface charge at the frictional pair–lubricant interface is wide, especially for the frictional pairs of ceramics which have numerous applications of water as a lubricant.

EDL is a physical structure spontaneously formed near the charged solid–liquid interface due to the electrostatic interaction between the charged interface and free ions within the liquid when the interface is charged due to different mechanisms [14–17]. To explain the effect of the EDL on the hydrodynamic lubrication, one of the fundamental mechanisms is the influence of the EDL on the apparent viscosity of the lubricant, which is referred as the electroviscous effect [17]. Although the applications and studies of lubrication theory have a history dating back to hundreds of years, the role of surface-charge-induced EDL on the lubrication is a relatively new field. This role was first considered for the study of thin film lubrication by Bike and Prieve [6]. Following them, Zhang and Umehara [7] introduced the effect of the EDL on modifying the conventional Reynolds equation, analyzing the hydrodynamic lubrication. They found that the minimum lubricant film thickness increased with the increasing absolute value of zeta potential (an important parameter of EDL to manifest the surface charge at the solid–liquid interface). Li [9] theoretically studied the combined roles of EDL and surface roughness on the hydrodynamic lubrication. Huang and his colleagues [8,10,13] systematically studied the effects of EDL on the hydrodynamic lubrication or elasto-hydrodynamic lubrication, where the effect of zeta potential was analyzed. All these studies revealed that the EDL can significantly affect the lubrication capacity, especially when the Debye length (an important parameter to manifest the thickness of the EDL) of the EDL is similar to the thickness of the lubricant film.

To investigate the role on the hydrodynamic lubrication played by the EDL, it is needed to obtain the electrical potential and ion concentration within the lubricant affected by the EDL. In the previous theoretical studies regarding the effect of the EDL on the hydrodynamic lubrication, the EDL-affected electrical potential and ion concentration were mainly obtained on the basis of solving the linear Poisson–Boltzmann equation (PBE), which is a simplified mathematical model under the assumption of the Debye–Hückel approximation (DHA) [6–11]. It should be noted that zeta potential must be strictly limited to a small range (normally, the magnitude of zeta potential should be smaller than 25 mV) in these analyses based on DHA, otherwise, an unallowable error can be introduced [17–22]. In such a small range of zeta potentials, the literature mainly revealed that the effect that the zeta potential has on lubrication, including minimum film thickness, pressure and load capacity, was a change in the form of a monotonic trend [6–11]. Actually, it was reported that the magnitude of zeta potential could be up to several hundred millivolts [23–25]. However, there are fewer studies regarding the dependence of the lubrication on the EDL with zeta potential (over a wide range) without the application of the DHA. Without the implementation of the DHA, Chakraborty and Chakraborty [12] carried out theoretical work to study the effect of the zeta potential in a range from −25. 9 to −77.6 mV on lubrication, with the additional consideration of the finite size of the ions. They found that the effect of the zeta potential on the load capacity resulted in a non-intuitive trend with increasing magnitude of zeta potential; however, further details and explanation were not given. In addition, the electroviscous effect is dependent on the electrical conductivity of the lubricant [26–27], which inevitably affects the hydrodynamic lubrication. However, most of the previous theoretical works neglected the variation of electrical conductivity of the lubricant considering the effect of the EDL.

To solve these problems, this paper presents a theoretical study on the effect of the EDL on the hydrodynamic lubrication with the additional consideration of the effect of the EDL on the electrical conductivity of the lubricant. The electrical potential and ion concentration distribution within the EDL are obtained by solving the nonlinear PBE without the use of the DHA. On the basis of these assumptions, the effect of the zeta potential on the apparent viscosity of the lubricant is first studied and analyzed. Then, by combining the effects of the EDL on the electrical conductivity and apparent viscosity of the lubricant, the conventional Reynolds equation is modified to study the effect of the EDL on the load capacity of hydrodynamic lubrication. In addition, the dependence of the apparent viscosity and load capacity on the bulk ion concentration of lubricant and lubricant film thickness are also investigated. The underlying mechanisms of these issues are analyzed.

## Theoretical Modeling

**Figure 1:**
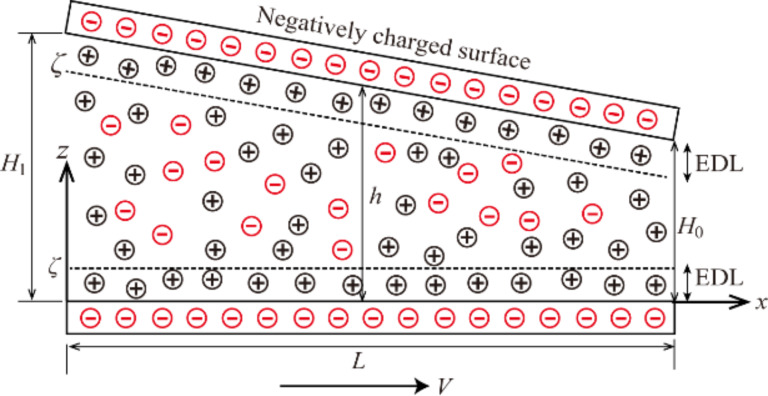
The schematic of hydrodynamic lubrication in a 1D slider bearing considering the effect of the EDL. *h* is a variable to indicate the lubricant film thickness, *H*_0_ and *H*_1_ are the lubricant film thickness at the outlet and inlet of bearing, respectively, *L* is the length of the bearing, *V* is the sliding velocity of the lower bearing surface, and *x* and *z* are the coordinate axes.

To analyze the effect of the EDL on the hydrodynamic lubrication, a hydrodynamic lubrication model in a 1D slider bearing with the effect of the EDL is considered. The physical model is shown in Figure 1. For the physical model in Figure 1, when the lubricant comes in contact with the slider bearing, two separate EDLs form near the upper solid–lubricant interface and the lower solid–lubricant interface. The following assumptions are made to carry out the modeling.

1. Both of these two bearing surface–lubricant interfaces are negatively charged and have the same zeta potential, ζ.
2. The lubricant film thickness is much larger than the Debye length, thus, the two EDLs are non-overlapped.
3. The no slip condition of the velocity is considered.
4. The lubricant is an incompressible Newtonian fluid in the steady laminar state. Inertial force is neglected when compared with viscous force.

To analyze the hydrodynamic lubrication under the effect of the EDL, the electrical potential within the EDL should first be analyzed. Normally, the nonlinear PBE is an effective tool to obtain the electrical potential within the EDL. However, it should be noted that the classical PBE is derived based on the assumption that the free ions in the lubricant are point-like. Under this assumption, the number density of the counter-ions adjacent to the interface can be infinitely large based on the PBE when the ion concentration and/or zeta potential is large enough, which is not valid for the actual situation. Thus, the steric effect has been proposed and introduced to correct the classical PBE [12,28–29]. Based on the previous studies, in a certain range of zeta potential whose magnitude is normally smaller than 300 mV for a dilute solution, the classical PBE can still effectively predict the electrical potential and ion concentration within an EDL with an allowable error when comparing with the prediction of theoretical model considering the steric effect [28]. Based on this analysis, in the present work, the PBE is still used to analyze the electrical potential distribution within the EDL. Furthermore, the zeta potential is strictly chosen during the simulation to reduce the error.

By solving the nonlinear PBE, the electrical potential φ of a non-overlapped EDL in the lubricant with a certain thickness of *h* can be derived as [12,17,27],

[1]
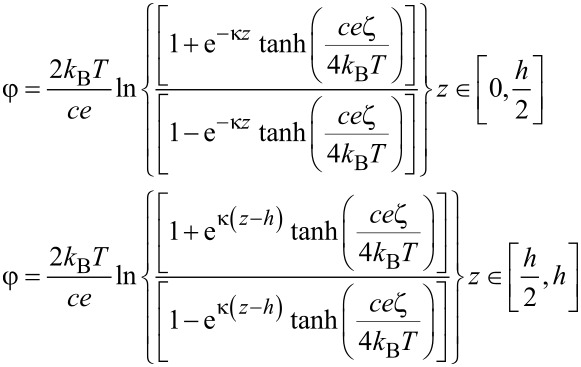


where κ = [2*n*_0_*c*^2^*e*^2^/(εε_0_*k*_B_T)]^1/2^ (*n*_0_ is the original bulk ion concentration of the lubricant, *c* is the chemical valence of free ions in the lubricant, *e* is the elementary charge, ε is the lubricant’s relative permittivity, and ε_0_ is vacuum’s absolute dielectric constant) is the reciprocal of the Debye length, *z* is the coordinate perpendicular to the lower bearing surface, ζ is the zeta potential at the solid–liquid interface and *h* is the lubricant film thickness.

By solving the modified Navier–Stokes equation describing the velocity field of the lubricant flowing in a 1D parallel plate channel with a height of *h* under the combined actions of the EDL, the driving pressure and the lower sliding wall with a velocity of *V*, the velocity field ν and the relevant volume flow rate of the lubricant *Q* can be derived as,

[2]
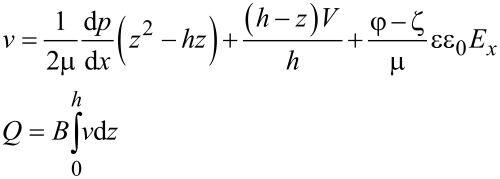


where μ is lubricant’s dynamic viscosity, d*p*/d*x* is pressure gradient, *V* is the sliding velocity of the lower surface, *E_x_* is the electrical field strength within the flowing EDL, and *B* is the width of the channel that is assumed to be much larger than the height of the channel.

For a steady lubricant flow, the electrical field strength *E_x_* can be obtained on the basis of when the total current within the lubricant is zero [17,30]. There are three kinds of electrical current induced by the EDL: the streaming current, the conduction current and the sliding-wall-induced current, which can be expressed in the following forms, respectively [17,30],

[3]
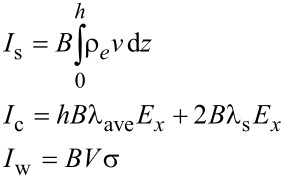


where *I*_s_ is the streaming current, *I*_c_ is the conduction current, *I*_w_ is the sliding-wall-induced current, ρ*_e_* = *ce*(*n*_+_−*n*_−_) is the local net ionic charge density within the EDL, λ_ave_ is the average bulk electrical conductivity of the lubricant, λ_s_ is the surface electrical conductivity of the lubricant that is neglected here, and σ = −εε_0_(dφ/d*z*)*_z_*_=0_ is the surface charge density at the lower bearing surface. Based on the zero net current in the steady state, the electrical field strength *E_x_* can be given as,

[4]
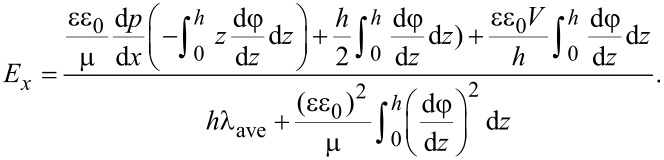


Based on the previous studies, the bulk electrical conductivity of the lubricant under the influence of the EDL increases with the increasing absolute value of the zeta potential [26–27]. Hence, the EDL-dependent electrical conductivity of the lubricant must be considered during the analysis of the electroviscous effect and hydrodynamic lubrication. The average bulk electrical conductivity under the influence of the EDL can be obtained as [26–27,31],

[5]
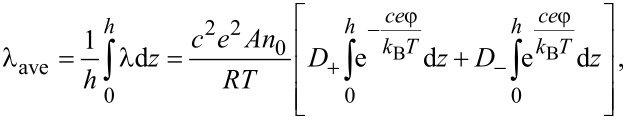


where *A* is Avogadro constant, *D*_+_ is counter-ion’s diffusion constant, *D*_−_ is co-ion’s diffusion constant, *R* is molar gas constant, *n*_+_ is counter-ion’s concentrations and *n*_−_ is co-ion’s concentration in the following equations.

[6]
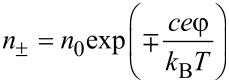


Normally, the electroviscous effect can be characterized by the apparent viscosity of the lubricant. Based on the apparent viscosity, the effect of the EDL on the flow can also be expressed by the following equation.

[7]
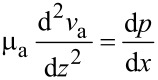


where μ_a_ is the lubricant’s apparent viscosity under the effect of the EDL, and ν_a_ is the apparent velocity field across the lubricant in the following equation,

[8]
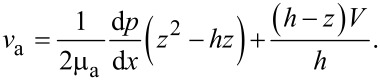


Using the apparent viscosity, the flow rate of the lubricant can be expressed as,

[9]
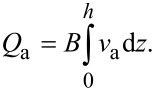


In practice, the flow rates in Equation 3 and Equation 8 are the same, thus, the apparent viscosity of the lubricant under the influence of the EDL can be expressed as,

[10]
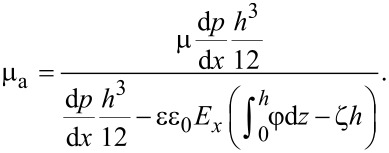


after obtaining the electrical potential within the EDL and apparent viscosity of the lubricant under the influence of the EDL. The effect of the EDL on the hydrodynamic lubrication can be derived as follows. Under the assumption of an incompressible lubricant, the mass conservation law gives the following modified Reynolds equation, which includes the effect of the EDL [7],

[11]
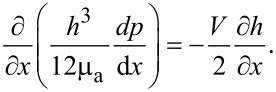


The hydrodynamic load capacity of the lubricant can be obtained by integrating the pressure over the 1D slider bearing length [7],

[12]
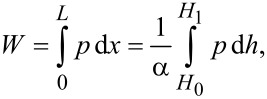


where *L* is the 1D slider bearing length, α is the slope of the upper bearing surface and *H*_0_ and *H*_1_ are the lubricant film thicknesses at the outlet and inlet of bearing, respectively. While solving for the hydrodynamic load capacity, the following pressure boundary conditions were used:

[13]
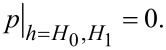


## Results and Discussion

After establishing the theoretical model regarding the effect of the EDL on the hydrodynamic lubrication, NaCl solution was chosen as the lubricant to carry out the analysis. The diffusion constants of Na^+^ and Cl^−^ are 1.334 × 10^−9^ m^2^/s and 2.032 × 10^−9^ m^2^/s, respectively [32]. To maintain the validity of the nonlinear PBE [28–29], the zeta potential is limited to a range from 0 mV to −250 mV.

### Electrical potential within the EDL

**Figure 2:**
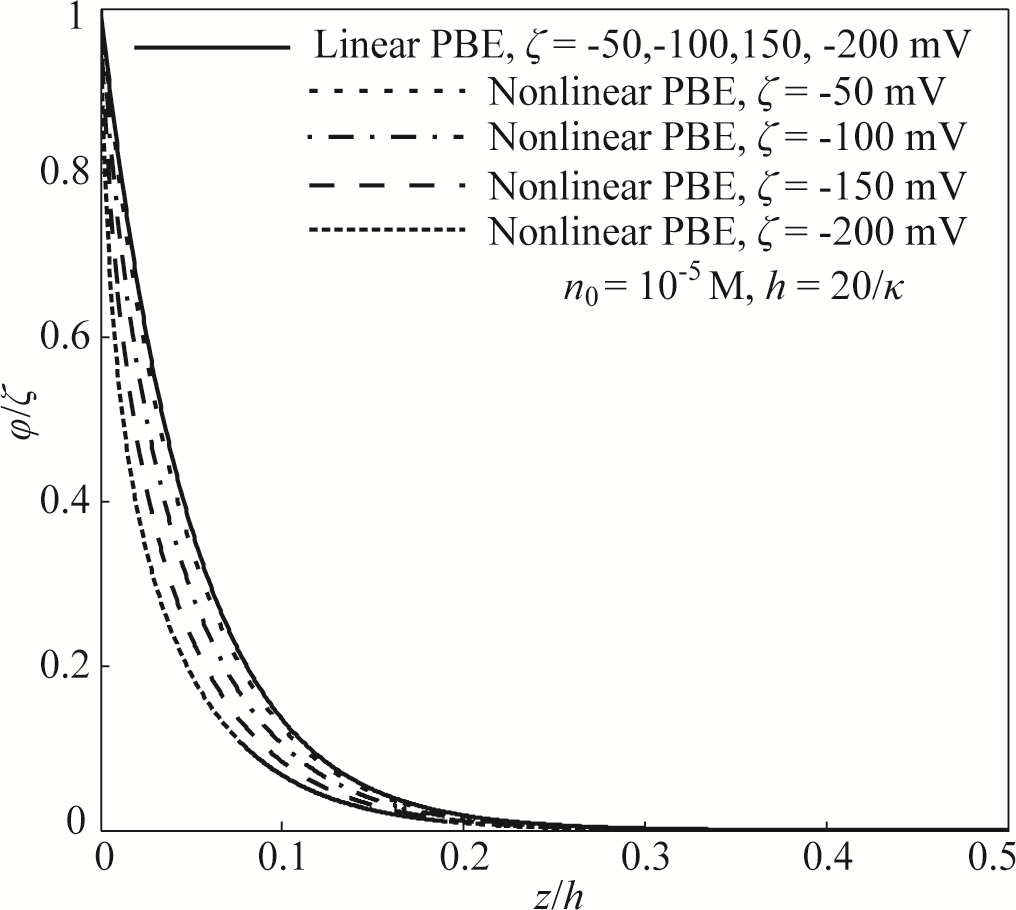
Dimensionless electrical potential distribution obtained by solving the linear PBE and the nonlinear PBE (the model used in present work).

Figure 2 shows the dimensionless electrical potential within the EDL as a function of dimensionless lubricant film thickness. To illustrate the differences induced by the DHA, the curves shown in Figure 2 are obtained by applying both the linear PBE and the nonlinear PBE, respectively. “Linear PBE” in the figure means the electrical potential is obtained on the basis of the linear PBE, while “Nonlinear PBE” means the electrical potential is obtained on the basis of the nonlinear PBE. Because the zeta potential at both the lower and upper bearing surfaces are the same, the electrical potential distribution within the EDL is symmetric. Hence, only half of the dimensionless electrical potential curves are given in Figure 2. It can be seen that the dimensionless electrical potentials obtained by the linear PBE remained constant with different values of zeta potential. Furthermore, there is a larger difference between the dimensionless electrical potentials obtained by the linear PBE and the nonlinear PBE when the zeta potential value is larger. Thus, the EDL-dependent lubrication analysis, based on the linear PBE, should be strictly limited to a small zeta potential range, otherwise, a large error will be introduced. To reduce this error when the magnitude of the zeta potential is large, the nonlinear PBE is used in present work to carry out the EDL-dependent lubrication analysis.

On the basis of the nonlinear PBE, Figure 2 shows that the dimensionless electrical potential decreases with an increase in the absolute value of the zeta potential. That is, the electrical potential shows a faster reduction. The mechanism for this phenomenon is described as follows. For the case of the zeta potential with a larger absolute value, there is a larger surface charge density at the bearing surface–lubricant interface. This results in a larger local net ionic charge density near the interface by attracting many more counter ions to close the interface, leading to a faster reduction of dimensionless electrical potential.

### Non-monotonic electroviscous effect

**Figure 3:**
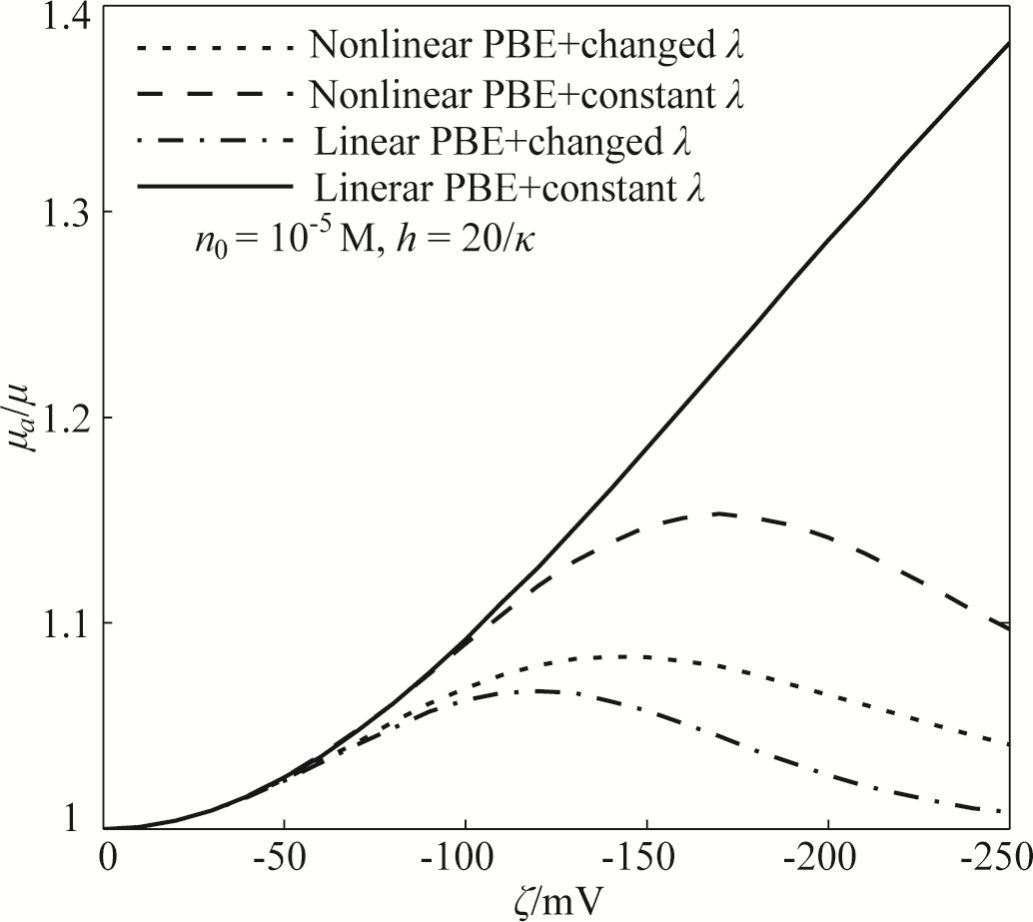
The effect of the zeta potential on the apparent viscosity of the lubricant based on different assumptions and theoretical models. The “Nonlinear PBE+changed *λ*” case is the model established in the present work.

Figure 3 shows variations of the EDL-dependent apparent viscosity of lubricant based on different assumptions and models. The label “changed *λ*” in the figure means that the variation of electrical conductivity with the zeta potential is considered during the analysis of apparent viscosity, while “constant *λ*” in the figure means that the influence of the zeta potential on the electrical conductivity is neglected during the analysis and the electrical conductivity is assumed to be a constant. From Figure 3, it can be found that there are large differences for the apparent viscosities of the lubricant based on different assumptions and models, especially when the absolute value of the zeta potential is large enough (up to hundreds of millivolts). When a constant electrical conductivity and the linear PBE based on DHA are used to analyze the effect of the zeta potential on the apparent viscosity, it can be found that the apparent viscosity of the lubricant monotonically increases with the increasing absolute value of the zeta potential. This holds even when the absolute value of the zeta potential is large (up to hundreds of millivolts), as seen by the solid line in Figure 3. This result is similar to the result reported by Li and Jin [11]. However, for the other three groups of results based on different assumptions, all of the three apparent viscosities show non-monotonic variations with the increasing absolute value of the zeta potential. Figure 3 shows that the theoretical model should be carefully established to guarantee the validity of EDL-induced apparent viscosity of the lubricant. It is believed that the theoretical model based on the nonlinear PBE and changed electrical conductivity is much more reasonable, and this is the model established and used in the present work.

**Figure 4:**
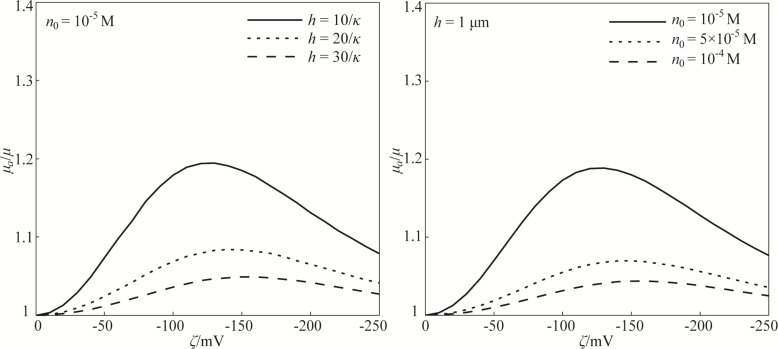
The effect of the zeta potential on the apparent viscosity of the lubricant and the dependence of the EDL-induced apparent viscosity on the lubricant film thickness and ion concentration of the lubricant based on the model of nonlinear PBE and modified electrical conductivity.

On the basis of the nonlinear PBE and modified electrical conductivity, Figure 4 shows the effect of the zeta potential of the EDL on the apparent viscosity of the lubricant and the dependence of the EDL-induced apparent viscosity on the lubricant film thickness and ion concentration of the lubricant. Figure 4 shows that the zeta potential leads to an increase in the apparent viscosity. More interestingly, the EDL-enhanced apparent viscosity shows a non-monotonic trend from increasing to decreasing with the gradually increasing absolute value of the zeta potential, that is, the electroviscous effect exhibits a non-monotonic characteristic.

The non-monotonic electroviscous effect has been systematically studied in our previous study and can be analyzed on the basis of the modified Navier–Stokes equation [27,33–35]. Based on the previous studies, the electrical body force applied on the flowing lubricant is dominated by the electrical field strength, which has a non-monotonic behavior with the increasing magnitude of the zeta potential. This non-monotonic electrical field strength leads to a non-monotonic electrical field force on the lubricant, which then results in a non-monotonic variation of the velocity and a non-monotonic electroviscous effect. In addition, the apparent viscosity increases with decreasing lubricant film thickness and decreasing ion concentration. These dependences of the electroviscous effect on the lubricant film thickness and ion concentration can also be explained on the basis of electrical field force applied on the lubricant, and have been studied in our previous works [27,33–35].

### Non-monotonic hydrodynamic load capacity of the lubricant

**Figure 5:**
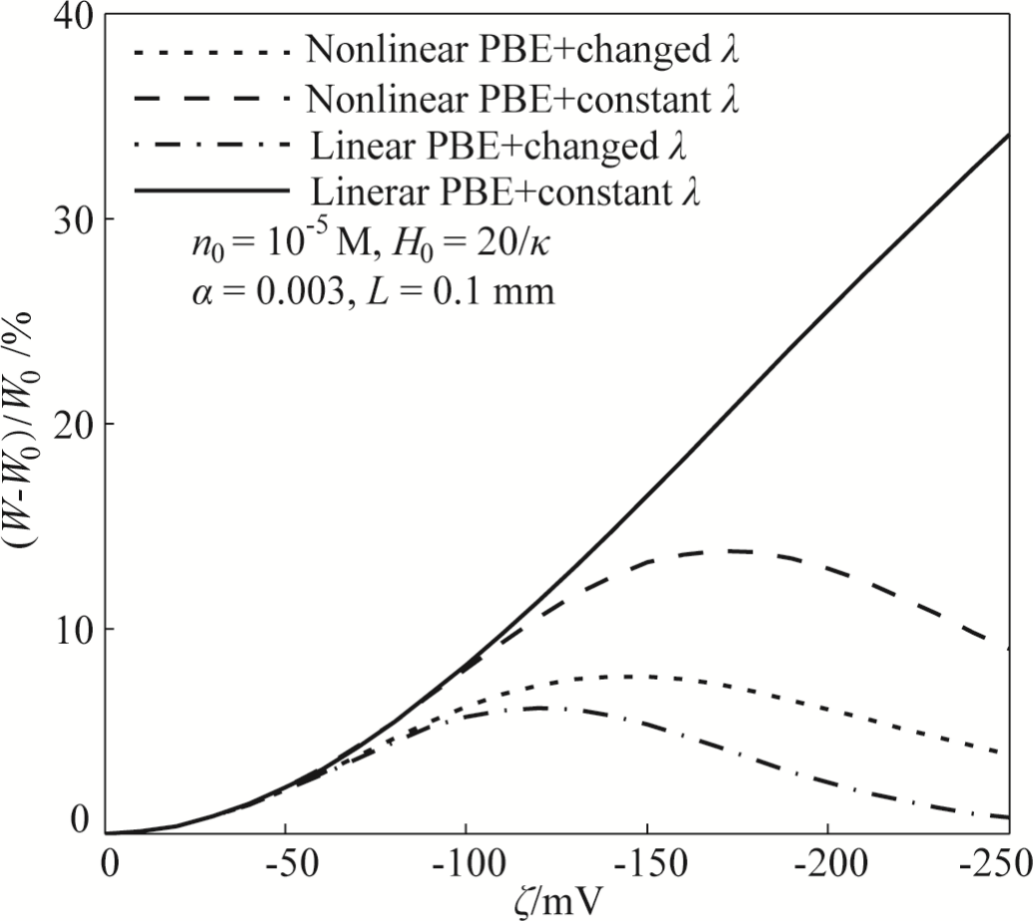
Comparison of the hydrodynamic load capacity of the lubricant including the effect of the zeta potential based on different assumptions and theoretical models. The “Nonlinear PBE+changed *λ*” is the model established and used in the present work.

Based on the influence of the EDL on the apparent viscosity, the effect of the EDL on the hydrodynamic load capacity of the lubricant can be analyzed. Figure 5 shows the effect of the EDL on the hydrodynamic load capacity of lubricant based on different theoretical models and different assumptions. *W*_0_ is the hydrodynamic load capacity without the effect of the zeta potential. *W* is the hydrodynamic load capacity with the effect of the zeta potential. Figure 5 shows that there are large differences for the hydrodynamic load capacity of the lubricant based on different models when the absolute value of the zeta potential is large enough. For example, when a constant electrical conductivity and the linear PBE are applied, the hydrodynamic load capacity of the lubricant monotonically increases with increasing absolute value of the zeta potential, even when the absolute value of the zeta potential is large. This result is similar to the reports of Li and Jin [11]. However, the other three types of hydrodynamic load capacities show non-monotonic variations with increasing zeta potential. The variation of the hydrodynamic load capacity is consistent with the variation of apparent viscosity. Among these models, the theoretical model based on the nonlinear PBE and varied electrical conductivity should be the most reasonable one, and this is the model used in the present work.

**Figure 6:**
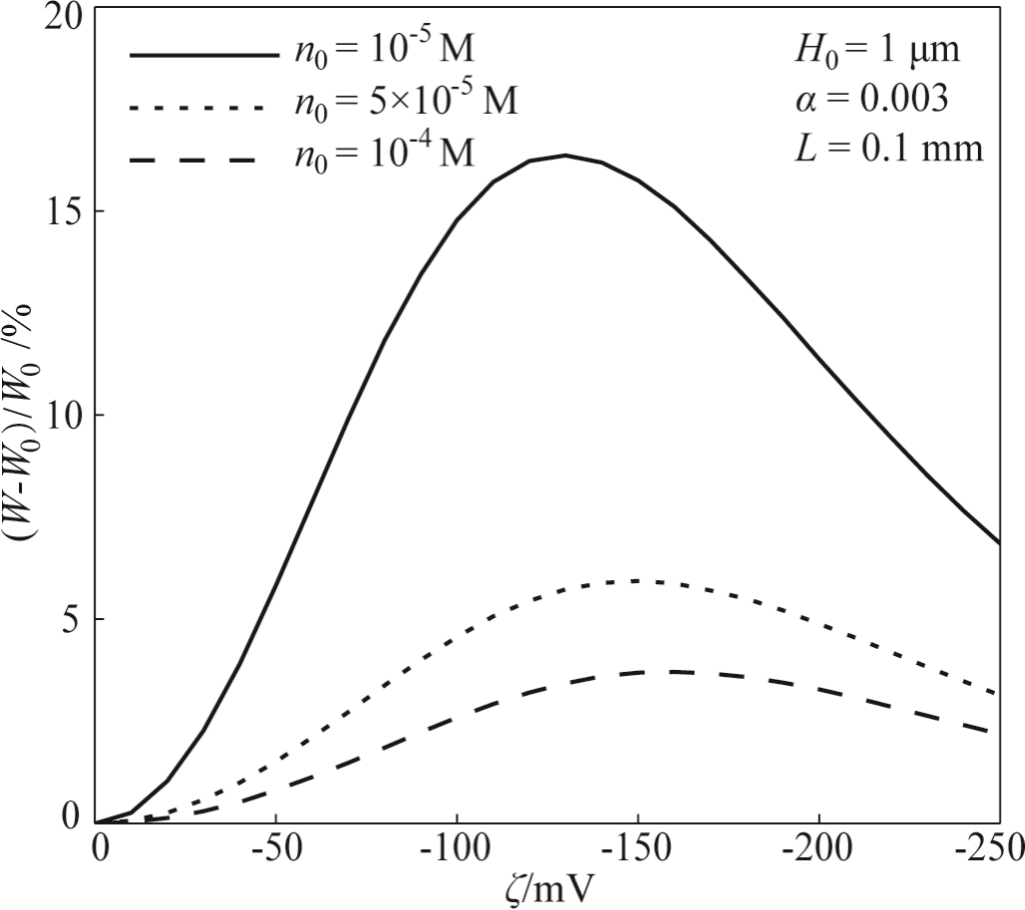
The effect of zeta potential on the hydrodynamic load capacity of the lubricant and the dependence of the hydrodynamic load capacity on the ion concentration based on the model of nonlinear PBE and varying electrical conductivity.

Figure 6 shows the influence of the zeta potential of the EDL on the hydrodynamic load capacity of the lubricant based on the nonlinear PBE and changed electrical conductivity. It can be concluded from Figure 6 that, although the EDL can enhance the hydrodynamic load capacity, the hydrodynamic load capacity shows a non-monotonic trend changing from enhancement to attenuation with the gradually increasing absolute value of the zeta potential. There is a non-monotonic relationship between the hydrodynamic load capacity and the zeta potential of the EDL. This result is similar to the result of Chakraborty and Chakraborty [12]. By considering the steric effect, they also found that the hydrodynamic load capacity followed a non-intuitive trend with increasing magnitude of zeta potential. The variation of the hydrodynamic load capacity with zeta potential is consistent with the variation of the apparent viscosity with zeta potential. A larger apparent viscosity leads to a stronger hydrodynamic load capacity. In addition, Figure 6 shows that the hydrodynamic load capacity increases with the decreasing ion concentration. This is because under the condition of a fixed lubricant film dimension, a smaller ion concentration of lubricant means a larger Debye length, and a larger range to affect the lubrication.

## Conclusion

In this paper, the effect of the zeta potential of the non-overlapped EDL on the hydrodynamic lubrication of a 1D slider bearing is theoretically studied on the basis of the nonlinear PBE without the use of the DHA. By considering the EDL-dependent bulk electrical conductivity, the effect of the EDL on the apparent viscosity of the lubricant was first studied to modify the classical Reynolds equation. Then, the effect of the zeta potential on the hydrodynamic lubrication was studied and analyzed based on the modified Reynolds equation.

The present study reveals that the apparent viscosity of the lubricant first increases and then decreases with the gradually increasing absolute value of zeta potential. The non-monotonic apparent viscosity including the effect of the zeta potential is dependent on the electrical field force applied on the lubricant, which is dominated by the non-monotonic electrical field strength. Being consistent with the variation in the apparent viscosity of the lubricant, the hydrodynamic load capacity of the lubricant under the effect of the EDL in a 1D slider bearing also shows a non-monotonic trend, changing from enhancement to attenuation with the gradually increasing absolute value of zeta potential. In addition, the hydrodynamic load capacity of the lubricant increases with decreasing ion concentration under the condition of fixed lubricant film thickness. Furthermore, the assumptions of DHA and constant electrical conductivity of the lubricant should be carefully applied to guarantee the validity of the hydrodynamic lubrication analysis.

The present work shows that the effect of a surface-charge-induced EDL on the hydrodynamic lubrication exhibits a non-monotonic behavior. The load capacity of a micro/nanometer sliding bearing firstly increases followed by a decrease with increasing absolute value of the zeta potential. Thus, an effective control of the surface charge or zeta potential at the bearing wall–lubricant interface can be a potential method to optimize the hydrodynamic lubrication capacity.
